# Investigating causal relations between sleep duration and risks of adverse pregnancy and perinatal outcomes: linear and nonlinear Mendelian randomization analyses

**DOI:** 10.1186/s12916-022-02494-y

**Published:** 2022-09-12

**Authors:** Qian Yang, Maria C. Magnus, Fanny Kilpi, Gillian Santorelli, Ana Gonçalves Soares, Jane West, Per Magnus, John Wright, Siri Eldevik Håberg, Eleanor Sanderson, Deborah A. Lawlor, Kate Tilling, Maria Carolina Borges

**Affiliations:** 1 MRC Integrative Epidemiology Unit, University of Bristol, Oakfield House, Oakfield Grove, Bristol, BS8 2BN UK; 2 Population Health Sciences, Bristol Medical School, University of Bristol, Bristol, UK; 3 Centre for Fertility and Health, Norwegian Institute of Public Health, Oslo, Norway; 4 Bradford Institute for Health Research, Bradford Teaching Hospitals NHS Foundation Trust, Bradford, UK; 5 National Institute for Health Research Bristol Biomedical Centre, University Hospitals Bristol NHS Foundation Trust and University of Bristol, Bristol, UK

**Keywords:** Sleep duration, Mendelian randomization, Pregnancy

## Abstract

**Background:**

Observational studies have reported maternal short/long sleep duration to be associated with adverse pregnancy and perinatal outcomes. However, it remains unclear whether there are nonlinear causal effects. Our aim was to use Mendelian randomization (MR) and multivariable regression to examine nonlinear effects of sleep duration on stillbirth (MR only), miscarriage (MR only), gestational diabetes, hypertensive disorders of pregnancy, perinatal depression, preterm birth and low/high offspring birthweight.

**Methods:**

We used data from European women in UK Biobank (*N*=176,897), FinnGen (*N*=~123,579), Avon Longitudinal Study of Parents and Children (*N*=6826), Born in Bradford (*N*=2940) and Norwegian Mother, Father and Child Cohort Study (MoBa, *N*=14,584). We used 78 previously identified genetic variants as instruments for sleep duration and investigated its effects using two-sample, and one-sample nonlinear (UK Biobank only), MR. We compared MR findings with multivariable regression in MoBa (*N*=76,669), where maternal sleep duration was measured at 30 weeks.

**Results:**

In UK Biobank, MR provided evidence of nonlinear effects of sleep duration on stillbirth, perinatal depression and low offspring birthweight. Shorter and longer duration increased stillbirth and low offspring birthweight; shorter duration increased perinatal depression. For example, longer sleep duration was related to lower risk of low offspring birthweight (odds ratio 0.79 per 1 h/day (95% confidence interval: 0.67, 0.93)) in the shortest duration group and higher risk (odds ratio 1.40 (95% confidence interval: 1.06, 1.84)) in the longest duration group, suggesting shorter and longer duration increased the risk. These were supported by the lack of evidence of a linear effect of sleep duration on any outcome using two-sample MR. In multivariable regression, risks of all outcomes were higher in the women reporting <5 and ≥10 h/day sleep compared with the reference category of 8–9 h/day, despite some wide confidence intervals. Nonlinear models fitted the data better than linear models for most outcomes (likelihood ratio *P*-value=0.02 to 3.2×10^−52^), except for gestational diabetes.

**Conclusions:**

Our results show shorter and longer sleep duration potentially causing higher risks of stillbirth, perinatal depression and low offspring birthweight. Larger studies with more cases are needed to detect potential nonlinear effects on hypertensive disorders of pregnancy, preterm birth and high offspring birthweight.

**Supplementary Information:**

The online version contains supplementary material available at 10.1186/s12916-022-02494-y.

## Background

Sleep occupies up to one third of the human life span. For adults, the minimum and maximum sleep durations are recommended as 7 and 9 h/day (h/d), respectively [1]. Habitual sleep duration is regulated by genetic factors [2] and can be influenced by a person’s daily routine and lifestyle factors [1]. Pregnancy is associated with alterations in sleep duration: both total and nocturnal sleep duration tend to be longer around the end of the first trimester declining by the third trimester due to pregnancy-induced changes, e.g. uterine contractions, heartburn, orthopnoea, leg cramps, pelvic girdle pain and uncomfortable sleeping position [3–5].

A systematic review of observational studies published up to 15 January 2018, which explored associations of prenatal sleep duration with psychological outcomes, reported a linear association of longer sleep duration with a lower risk of perinatal depression [6]. Other systematic reviews indicate that both short (<6 or <7 h/d) and/or long (>9 h/d) duration are associated with higher risks of adverse perinatal events (see Additional file 1: Table S1) [6–13]. In particular, short sleep duration is associated with higher risks of gestational diabetes [7, 8], preeclampsia [8] and preterm birth [8–10], while long sleep duration is associated with higher risks of stillbirth [8, 11] and gestational diabetes [8, 12]. These observational studies may be vulnerable to residual confounding, with demonstrated between-study heterogeneity likely influenced by variation in confounder control [14]. Few studies have examined several outcomes together, which is important for trying to identify a range of duration that minimizes any adverse outcomes. Studies to date have mostly examined binary variables of short and long sleep duration rather than trying to explore different patterns across sleep duration.

In the absence of large, well-conducted randomized controlled trials of interventions targeting on sleep duration during pregnancy [15], Mendelian randomization (MR) provides an alternative means of probing the effect of sleep duration on adverse pregnancy and perinatal outcomes. MR uses single nucleotide polymorphisms (SNPs) that are robustly associated with potential risk factors, e.g. sleep duration, as instrumental variables (IVs) to explore causal effects of these factors on outcomes [16, 17]. MR is less prone to confounding than observational studies, as SNPs being randomly allocated at meiosis cannot be influenced by the wide range of socio-demographic or behavioural factors conventionally confounding observational studies, nor can they be influenced by health status [16, 17]. Under key assumptions (see the “Discussion” section), MR can be used to estimate a causal effect from SNPs-risk factor and SNPs-outcome associations, and to estimate nonlinear relationships [18, 19]. SNPs robustly associated with self-report sleep duration have recently been identified in the most updated genome-wide association study (GWAS) using UK Biobank (UKB) [2]. These SNPs have been used as IVs in MR studies to investigate linear and nonlinear effects of sleep duration on cancer [20], cardiometabolic health [21, 22] and mental health [23, 24]. To the best of our knowledge, MR has not been used to explore effects of sleep duration on pregnancy and perinatal outcomes.

Our aims are to use MR to explore and compare linear and nonlinear effects of lifelong sleep duration on partum-related (stillbirth, miscarriage and preterm birth), pregnancy-related (gestational diabetes, hypertensive disorders of pregnancy and perinatal depression) and offspring-related (low birthweight, high birthweight and variation in birthweight) outcomes in up to 324,826 women. We also conducted confounder-adjusted linear and nonlinear multivariable regression (MVreg) of maternal sleep duration reported during pregnancy with outcomes, except stillbirth and miscarriage, in 76,669 women.

## Methods

### Participants

This study was undertaken using data from the MR-PREG collaboration, which aims to explore causes and consequences of different pregnancy and perinatal events [25]. We include women of European descent from (1) UKB (176,897 women recruited at age 40–60 years between 2006 and 2010 and providing retrospective reports of pregnancy and perinatal outcomes (Additional file 2: Fig S1A) [26]); (2) FinnGen (the nation-wide network of Finnish biobank with up to 123,579 women recruited at 54 years (interquartile range=25) with outcomes obtained via health record linkage), and three birth cohorts (women recruited during pregnancy with most outcomes collected prospectively); (3) Avon Longitudinal Study of Parents and Children (ALSPAC, 6826 women recruited between 1991 and 1992 (Additional file 2: Fig S1B)); (4) Born in Bradford (BiB, 2940 women recruited between 2007 and 2010 (Additional file 2: Fig S1C)); and (5) the Norwegian Mother, Father and Child Cohort Study (MoBa, 76,669 women in MVreg, of whom 14,585 women had genome wide data and were included in two-sample MR; recruited between 1999 and 2009 (Additional file 2: Fig S1D)). All studies had ethical approval from relevant national or local bodies and participants provided written informed consent. Details of their recruitment procedures, information on genetic data and measurements of baseline characteristics are described in Additional file 2: Text S1 [23, 25–41].

### Self-report sleep duration

**Fig. 1:**
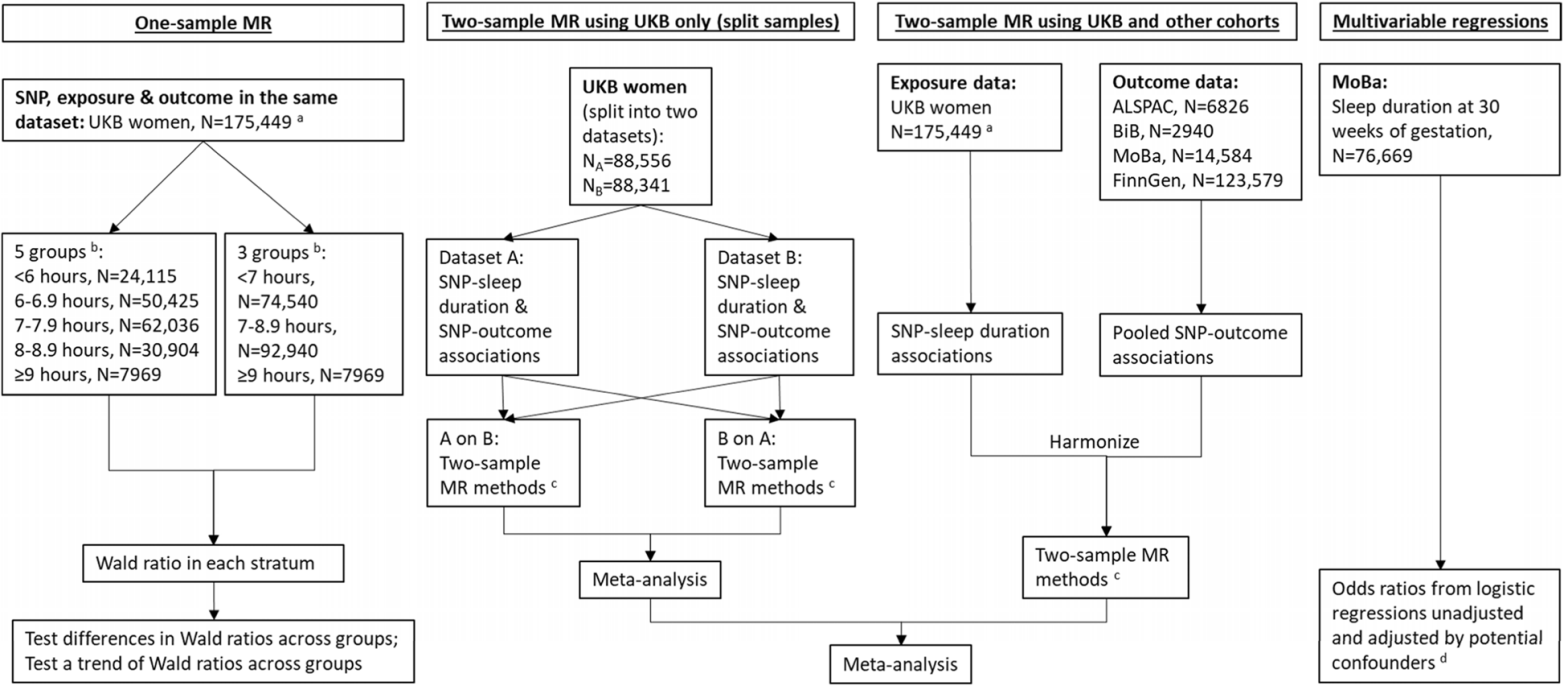
Summary of methods and data contributing to this study. ^a^Among 176,897 women, 99.2% of them with sleep duration ranging from 2 to 12 h were included. ^b^In one-sample MR, the three and five groups of different duration lengths are based on thresholds from existing literature [1, 8]. We also split UKB women into thirds (*N*=58,483) as a sensitivity analysis to increase instrument strength and power in the longest duration group. ^c^Two-sample MR methods include: inverse variance weighted, MR-Egger, weighted median and leave-one-out analysis. ^d^Multivariable regression analysis adjusted for maternal age at time of delivery, parity, education, smoking status in pregnancy, alcohol intake in pregnancy, body mass index before pregnancy and average household income. Abbreviations: ALSPAC, Avon Longitudinal Study of Parents and Children; BiB, Born in Bradford; MoBa, Norwegian Mother, Father and Child Cohort Study; MR, Mendelian randomization; SNP, single nucleotide polymorphism; UKB, UK Biobank

Information on sleep duration was obtained from UKB and MoBa. In UKB, sleep duration was measured via a self-administered question—“About how many hours sleep do you get in every 24 hours? (please include naps)” at the initial assessment centre, which recruited mostly non-pregnant participants. Women reported their sleep duration in integer values ranging from 1 to 23. Following the methods of a previous MR study [23], 1448 (0.8%) women with sleep duration shorter than 2 h or longer than 12 h were treated like those who had not responded to this question. UKB contributed to two-sample (linear) and one-sample (nonlinear) MR (Fig. 1).

In MoBa, sleep duration was assessed via a self-administered question—“How many hours a day do you usually sleep now when you are pregnant?” at 30 weeks of gestation. Women reported their sleep duration in five categories, which were “over 10 h”, “8–9 h”, “6–7 h”, “4–5 h” and “less than 4 h”. The questionnaire did not specify whether to include naps so it is unclear whether the women would have reported duration only for night sleep or across 24 h (as in UKB). Due to small numbers, we combined the last two categories into “≤5 h”. MoBa contributed to analyses of the relevance of genetic IVs in pregnancy, two-sample MR and linear/nonlinear associations in MVreg (Fig. 1).

Sleep duration data were not available in ALSPAC, BiB or FinnGen, which all contributed to two-sample MR only (Fig. 1).

### Selection of genetic IVs for self-report sleep duration

Currently, nine GWAS of self-report sleep duration are available, with details in Additional file 1: Table S2 [2, 42–51]. All GWAS combined women and men (mainly of White European descent) with no sex specific analyses; the four largest GWAS (*N* >100,000) all included UKB. The largest and most updated GWAS identified 78 SNPs genome-wide significantly (*P*-value<5×10^−8^) associated with sleep duration in its discovery cohort—UKB men and women (*N*=446,118), with 55 of them being directionally consistent in the replication cohort—the Cohorts for Heart and Aging Research in Genomic Epidemiology (CHARGE, *N*=47,180) [2]. To maximize statistical power and IV strength in our main analyses, we considered the 78 discovery SNPs from this largest GWAS. Additional file 1: Table S3 lists the characteristics of these 78 SNPs, as well as summary data for their association with sleep duration in UKB women used in this study. The 78 SNPs explained 0.69% of the variance in sleep duration in UKB [2]. We used the “clumping” function from the TwoSample MR R package [52], to check that the 78 SNPs were independent (i.e. linkage equilibrium) based on a threshold of *R*^2^≤0.01 and all European samples from the 1000 genome project as the reference population; the 78 SNPs are all independent of each other. For two-sample MR, we used these 78 SNPs as IVs with their effect alleles identified from the GWAS discovery results. For one-sample MR, we combined these 78 SNPs with the same effect alleles into an unweighted genetic risk score [53]. It was not possible to obtain external (to UKB) weights from genetic association estimates generated in the GWAS discovery analyses (due to sample overlap with our analyses sample) or replication stage (due to the trait increasing allele being inconsistent between discovery and replication stages for some SNPs).

The same GWAS has also reported results for short (defined as ≤6 h/d) and long (defined as ≥9 h/d) sleep duration, identifying 27 and 9 independent genome-wide significant SNPs, respectively [2]. We decided a priori not to use these in the one-sample MR in order to explore other possible nonlinear association or different thresholds of “healthy” sleep duration to these and for different outcomes. There were further technical considerations that are described in more detail in Additional file 2: Text S2 [2, 24, 54–56].

### Pregnancy and perinatal outcomes

We examined associations with nine outcomes. Partum-related outcomes included stillbirth, miscarriage and preterm birth (gestational age <37 completed weeks). Pregnancy-related outcomes included gestational diabetes, hypertensive disorders of pregnancy and perinatal depression (occurring during pregnancy or within a year after delivery). Offspring-related outcomes included low birthweight (birthweight <2500 g), high birthweight (birthweight >4500 g) and birthweight (grams) as a continuous outcome. Definitions of these outcomes in UKB, ALSPAC, BiB, MoBa and FinnGen, and harmonization of their definitions across cohorts are provided in Additional file 1: Table S4. In UKB, women who reported both never experiencing a pregnancy loss and giving birth to zero child were defined as never pregnant and thus removed from the analyses. If multiple pregnancies were enrolled in the birth cohorts, we randomly selected one pregnancy per woman. In FinnGen, it was only possible to include miscarriage (*N*=9113 cases/89,340 controls), gestational diabetes (*N*=5687 cases/117,892 controls), hypertensive disorders of pregnancy (*N*=4255 cases/114,735 controls) and preterm birth (*N*=5480 cases/98,626 controls), and those outcomes were defined based on ICD codes [39].

We combined pre-eclampsia and gestational hypertension as hypertensive disorders of pregnancy since we would not have sufficient statistical power to consider pre-eclampsia separately. In UKB, gestational age was only available for a subset of women (*N*=7280) who were young enough to have had a child born during or after 1989, the earliest date for which linked hospital labour and perinatal data are available. As a result, numbers with data on preterm birth are smaller than for any other outcome, and we decided a priori to examine associations with low/high birthweight rather than small-/large-for-gestational age. In the three birth cohorts, stillbirth and miscarriage were retrospectively obtained (from self-report or clinical records) at the time of the index pregnancy. In ALSPAC and MoBa, women were asked if they had ever experienced a (previous) stillbirth or miscarriage, but numbers of pregnancy loss at the index pregnancy were too small for reliable results. Additionally, for miscarriage, we were concerned about misclassification or selection bias due to women who had experienced a miscarriage prior to recruitment.

### Confounders in MoBa for MVreg

We considered maternal age at time of delivery, parity, education, smoking status in pregnancy, alcohol intake in pregnancy, body mass index before pregnancy and average household income as potential confounders based on their known or plausible effects on maternal sleep duration and on pregnancy and perinatal outcomes. Details of how these variables were measured are provided in Additional file 2: Text S1.

### Statistical analyses

#### One-sample MR exploring whether data support nonlinear over linear effects

One-sample MR requires individual level data for estimation, and thus, we used data from UKB women (Fig. 1). Nonlinear MR involves generating subgroups of different sleep duration length within the study sample and undertaking (linear) MR within each of those subgroups and then comparing effects across subgroups. We could split the women into subgroups based on their reported sleep duration, but doing that could introduce a type of selection bias known as collider bias in the subsequent MR, because of the role of the genetic IV on sleep duration [19]. To avoid that, we generated “residual” sleep duration by regressing self-reported sleep duration on the genetic risk score, adjusting for genetic array, women’s age and top 40 PCs (to adjust for residual population stratification [57]). Residual sleep duration was then calculated as each woman’s observed sleep duration minus the mean centred genetic contribution to sleep duration from the IV [19, 55]. Therefore, the residual measure has a mean of 7.18 with a range from 1.77 to 12.33 h/d (Additional file 2: Fig S2) [55]. We then stratified UKB women into three and five groups based on the residual duration (details shown in Additional file 1: Table S5). We present results for three and five groups and compared effects of increasing sleep duration (measured in 1-h units) from MR analyses across the groups. Three groups would enable comparing results between analyses with greater power and IV strength, while five groups provide finer gradation for more detailed exploration of nonlinearity.

Within each subgroup of residual sleep duration, we followed the approach used in a previous MR study [55] to calculate linear MR estimates for sleep duration on pregnancy and perinatal outcomes using the Wald ratio method [56]. Technical details of this method are described in Additional file 2: Text 2. Finally, we tested differences in MR estimates across groups using Cochran’s *Q*-statistic, with *P*-value <0.05 suggesting heterogeneity [19, 55]. We performed meta-regression of MR estimates against the mean of sleep duration in each group to test nonlinearity [19, 55]. A low *P*-value for the regression coefficient of sleep duration mean across groups provides evidence against the null hypothesis of a linear or null association, and we used the conventional *P*-value <0.05 as evidence to support nonlinearity. Similar non-null effects across the group (e.g. if there was evidence of a similar magnitude positive effect in all groups) would support a linear effect. Table 1 illustrates how the pattern of a nonlinear effect is identified by comparing the magnitudes and directions of linear associations in each group and synthesizing results from one-sample and further two-sample MR.

**Table 1:**
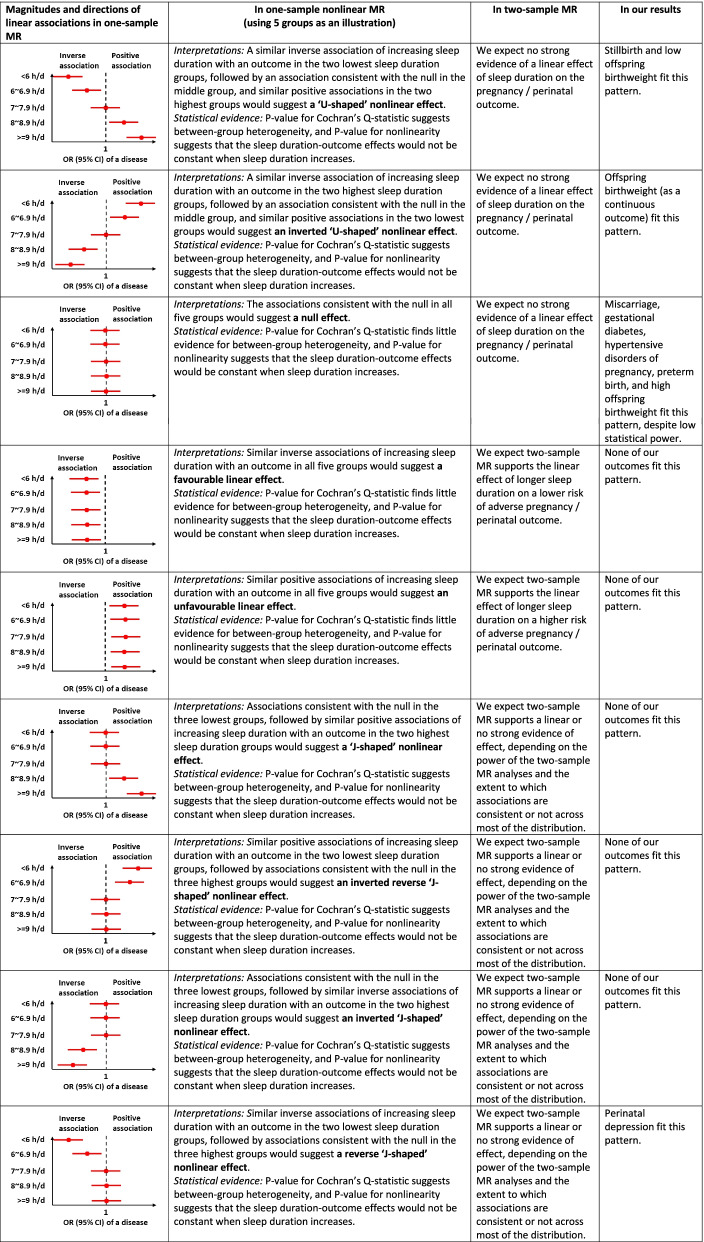
Possible patterns of effects identified by one-sample nonlinear MR and two-sample MR

*CI* confidence interval, *MR* Mendelian randomization, *OR* odds ratio

#### Two-sample MR exploring linear effects

We further undertook two-sample MR to explore potential linear effects (Fig. 1). Details about obtaining SNP-sleep duration and SNP-outcome associations are described in Additional file 2: Text S1. In UKB women, we generated summary statistics from the individual participant data in a split cross-over samples design [23]. This involved randomly splitting the sample in half and generating summary data for SNP-sleep duration and SNP-outcome associations in both datasets and then conducting MR with SNP-sleep duration associations from dataset A and SNP-outcome associations from dataset B, and vice versa [23]. This was because the GWAS of sleep duration was conducted in UKB [2], and this split cross-over design enabled us to have the advantages (e.g. weak instrument bias towards the null and minimizing over-prediction or winners curse) of MR using two independent samples [30, 58]. We then meta-analysed the MR estimates from the two together for each sleep duration-outcome pair using fixed-effects (with inverse variance weights). We also conducted two-sample MR using SNP-sleep duration associations from UKB women, and SNP-outcome associations meta-analysing ALSPAC, BiB, MoBa and FinnGen using fixed effects with inverse variance weights. For each outcome, we combined MR estimates from all five cohorts using fixed effects (with inverse variance weights). The degree of between-study heterogeneity was assessed using Cochran’s Q-statistic.

We used the MR inverse variance weighted (IVW) method as the main analysis to explore the presence of linear effects of sleep duration on pregnancy and perinatal outcomes. IVW is a weighted regression of SNP-outcome associations on SNP-sleep duration associations with the intercept of the regression line forced through zero [59].

#### Sensitivity and additional analyses

The strength of the IVs was evaluated by the *F*-statistic of IV-sleep duration associations [17]. We selected the 78 SNPs robustly related to sleep duration in general population rather than pregnant women [2]. Therefore, we used linear regression to test whether our IV was also related to sleep duration during pregnancy in MoBa [16, 17]. The one-sample MR assumes the genetic IV-sleep duration association is consistent across groups [19, 55]. We explored this by using Cochran’s Q-statistic and meta-regression of these associations against the mean of observed sleep duration in each stratum [19, 55].

As with previous two-sample MR studies testing effects of sleep duration on different outcomes [20–24], we used the 78 genome-wide significant SNPs from the discovery sample in the original GWAS to maximize power and IV strength. To explore whether our results were sensitive to IV selection, we repeated IVW analyses with the 55 SNPs that were directionally consistent in the replication sample and the 43 SNPs of those that also reached genome-wide significance. To explore potential unbalanced horizontal pleiotropy, our sensitivity analyses for two-sample MR included (I) assessing between-SNP heterogeneity (which if present may be due to one or more SNPs having horizontal pleiotropic effects on the outcome) using Cochran’s Q-statistic and leave-one-out analysis [59] and (II) conducting weighted median [60] and MR-Egger [61], which are more likely to be more robust in the presence of horizontal pleiotropy [62]. Technical details of these MR methods were summarized in our previous study [63]. A consistent finding across multiple MR methods would strengthen causal inference. When using MR to assess maternal exposures in pregnancy on perinatal outcomes, results might be biased via a path from maternal genotype to the outcome via fetal genotype [64]. To explore this, we compared maternal SNP-outcome associations with and without adjustments for fetal SNPs in the birth cohorts.

#### MVreg in MoBa exploring linear/nonlinear associations and whether data supported nonlinear over linear associations

We explored observational associations of maternal sleep duration at 30 weeks of gestation with each outcome, except stillbirth and miscarriage, using logistic regression (linear regression for birthweight). To explore a possible nonlinear association, we entered the categories as indicator variables and obtained estimates comparing each of ≤5 h/d, 6–7 h/d and ≥10 h/d to our chosen reference category of 8-9 h/d. We also explored possible linear associations by recoding ≤5, 6–7, 8–9 and ≥10 h/d categories using their mid-points (i.e. 3.5, 6.5, 8.5, 11 h/d, respectively), assuming MoBa had the same minimum and maximum sleep duration as UKB. Statistical evidence for a nonlinear association across categories was obtained from a likelihood ratio test comparing the two models above.

Amongst the 76,669 MoBa women eligible for inclusion in MVreg (defined as having returned both pregnancy questionnaires [65]), there were varying amounts of missing data for sleep duration, outcomes and covariates. This was lowest for parity (0.2% missing), and highest for preterm birth (8.4% missing). Additional file 1: Table S6 provides full details of the proportion missing for each variable. Therefore, we undertook both complete records and multiple imputation analyses. Complete records analyses only included women with sleep duration, an outcome and seven confounders (*N*=42,001 (for stillbirth) to 62,929 (for birthweight)), assuming that missingness is not associated with the outcome. MI was conducted on all 76,669 eligible women, and assumes data are missing at random (i.e. conditional on variables included in multiple imputation, the outcome would not differ between those with missing data and those without) [66, 67].

Multiple imputation used chained equations [66] and was conducted for each outcome separately. As shown in Additional file 1: Table S6, each imputation model included one outcome, the exposure (sleep duration), the seven confounders (same as those used in complete records analyses) and three auxiliary variables (paternal education, paternal smoking status in pregnancy and maternal usage of other kinds of nicotine in pregnancy). These auxiliary variables were selected on the basis that they were likely to be important predictors of missing data. For each outcome, 100 imputed datasets were generated and results were pooled across these datasets using Robin’s Rules [68].

All multiple imputation and MVreg after imputation were conducted in Stata 16 (StataCorp LLC, College Station, TX), because we were using code provided in the STRengthening Analytical Thinking for Observational Studies (STRATOS) framework for dealing with missing data and this is only provided in Stata [66]. All other analyses (including one- and two-sample MR, and complete records MVreg analyses) were conducted in R version 3.5.1 (R Foundation for Statistical Computing, Vienna, Austria), with “TwoSampleMR” package for two-sample MR [52].

## Results

Table 2 summarizes the characteristics of included women for MR analyses, and the proportion of cases for pregnancy and perinatal disorders across the four cohorts, which differ substantially for some outcomes. Additional file 1: Table S6 summarizes the characteristics of MoBa women for MVreg analyses.

**Table 2:** Characteristics of the women in UK Biobank, ALSPAC, BiB and MoBa included in Mendelian randomization

Variable ^**a**^	UK Biobank (***N***=176,897)	ALSPAC (***N***=6826)	BiB (***N***=2940)	MoBa (***N***=14,584)
	*Mean (standard deviation)*			
Maternal age (years)	25.5 (4.6) ^b^	28.7(4.7)	26.8 (6.0)	30.0 (4.5)
Maternal height (cm)	162.5 (6.2)	164.3 (6.7)	164.4 (6.1)	168.4 (5.7)
Maternal body mass index (kg/m^2^)	27.0 (5.1)	22.9 (3.7)	26.7 (6.0)	24.0 (4.2)
Parity	2.2 (1.0)	0.8 (0.9)	0.8 (1.1)	0.7 (0.8)
Gestational age (weeks)	38.9 (3.8) ^c^	39.6 (1.7)	39.7 (1.9)	39.6 (1.7)
Offspring birthweight (grams)	3186.7 (547.6)	3441.5 (523.0)	3357.9 (571.2)	3640.8 (513.4)
	*N (%)*			
Maternal education				
O levels/GCSEs or equivalent and below	81,113 (46.4)	4043 (59.5)	1400 (47.6)	252 (1.7)
A levels/AS levels or equivalent	40,817 (23.3)	1719 (25.3)	485 (16.5)	4378 (30.0)
College or university degree	53,062 (30.3)	1035 (15.2)	551 (18.7)	9100 (62.4)
Maternal ever smoking	74,308 (42.2)	1450 (21.6) ^d^	911 (31.0) ^d^	1106 (7.6) ^d^
Maternal ever drinking	162,436 (92.0)	4580 (70.2) ^d^	1793 (61.0) ^d^	3644 (25.0) ^d^
Offspring sex, male	Not available	3430 (50.2)	1504 (51.2)	7456 (51.1)
Number with fetal genotype data	0	4625 (67.8)	1855 (63.1)	12,183 (83.5)
	N cases / N controls (Proportion, %)			
Stillbirth	4907/107,791 (4.4)	48/4546 (1.0)	31/2588 (1.2)	51/9998 (0.5)
Miscarriage	42,717/107,791 (28.4)	1378/4546 (23.3)	14/2588 (0.5)	2677/9998 (21.1)
Gestational diabetes mellitus	726/170,308 (0.4)	34/6283 (0.5)	136/2657 (4.9)	113/14,375 (0.8)
Hypertensive disorders of pregnancy	2128/174,769 (1.2)	1099/5698 (16.2)	347/2159 (13.8)	1892/12,652 (13.0)
Perinatal depression	5168/20,860 (19.9)	423/5896 (6.2)	312/2245 (12.2)	579/13,865 (4.0)
Preterm birth	551/4811 (10.3) ^c^	285/4931 (5.5)	172/2706 (6.0)	495/12,846 (3.7)
Low offspring birthweight	13,429/149,084 (8.3)	337/6376 (5.0)	167/2725 (5.8)	245/13,690 (1.8)
High offspring birthweight	2716/149,084 (1.8)	113/6376 (1.7)	42/2725 (1.5)	621/13,690 (4.3)

^a^In UKB, these variables were measured at the recruitment that is typically many years after pregnancy

^b^We report maternal ages at giving their first live birth. UK Biobank women were recruited with an average age of 56.9 (standard deviation 7.8) years

^c^Gestational age was available only in a small subset of UK Biobank women (*N*=7280)

^d^These were maternal ever smoking/drinking in pregnancy

*ALSPAC* Avon Longitudinal Study of Parents and Children, *BiB* Born in Bradford, *MoBa* Norwegian Mother and Child Cohort Study

### One sample MR in UKB exploring whether data supported nonlinear over linear effects

The *F*-statistic for our genetic risk score in 175,499 UKB ever pregnant women was 1219, and within each group across all three ways in which they were stratified were between 55 and 646 (Additional file 1: Table S5). For each stratification approach, we also identified statistical evidence for between-group differences in associations of genetic risk score with sleep duration (Additional file 1: Table S5).

**Fig. 2:**
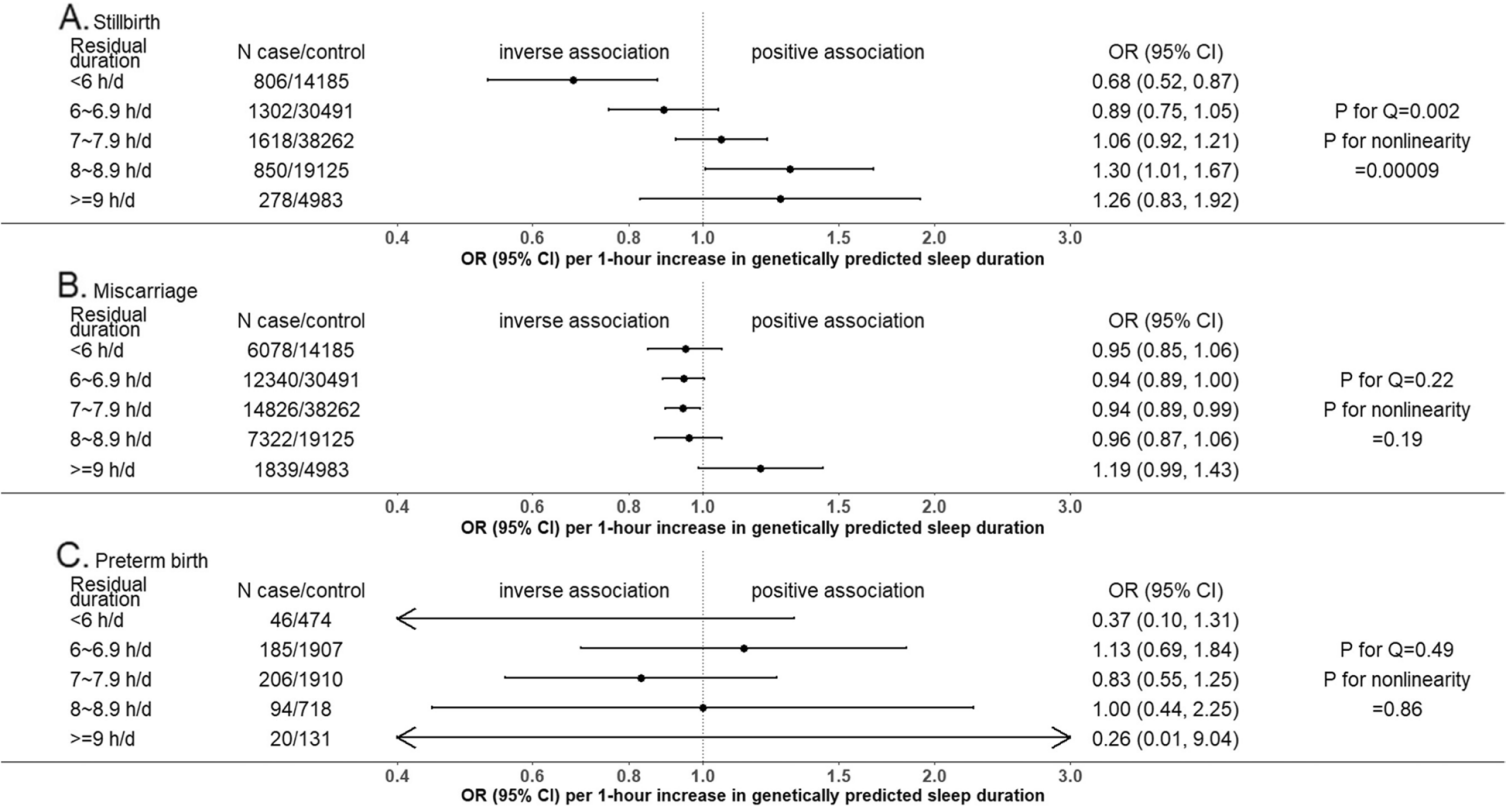
One-sample MR estimates of effects of sleep duration on partum-related outcomes in five groups in UK Biobank women. We present MR estimates of a linear effect of increasing duration on the outcome across the length of residual duration (h/day) covered in each group. Further details about identifying the pattern of nonlinear effects are illustrated in Table 1. *P*-value for Cochran’s *Q*-statistic testing statistical evidence for between-group heterogeneity. *P*-value for nonlinearity testing statistical evidence whether the MR estimates are changed as the self-reported sleep duration mean increases. Abbreviation: CI, confidence interval; MR, Mendelian randomization; OR, odds ratio

**Fig. 3:**
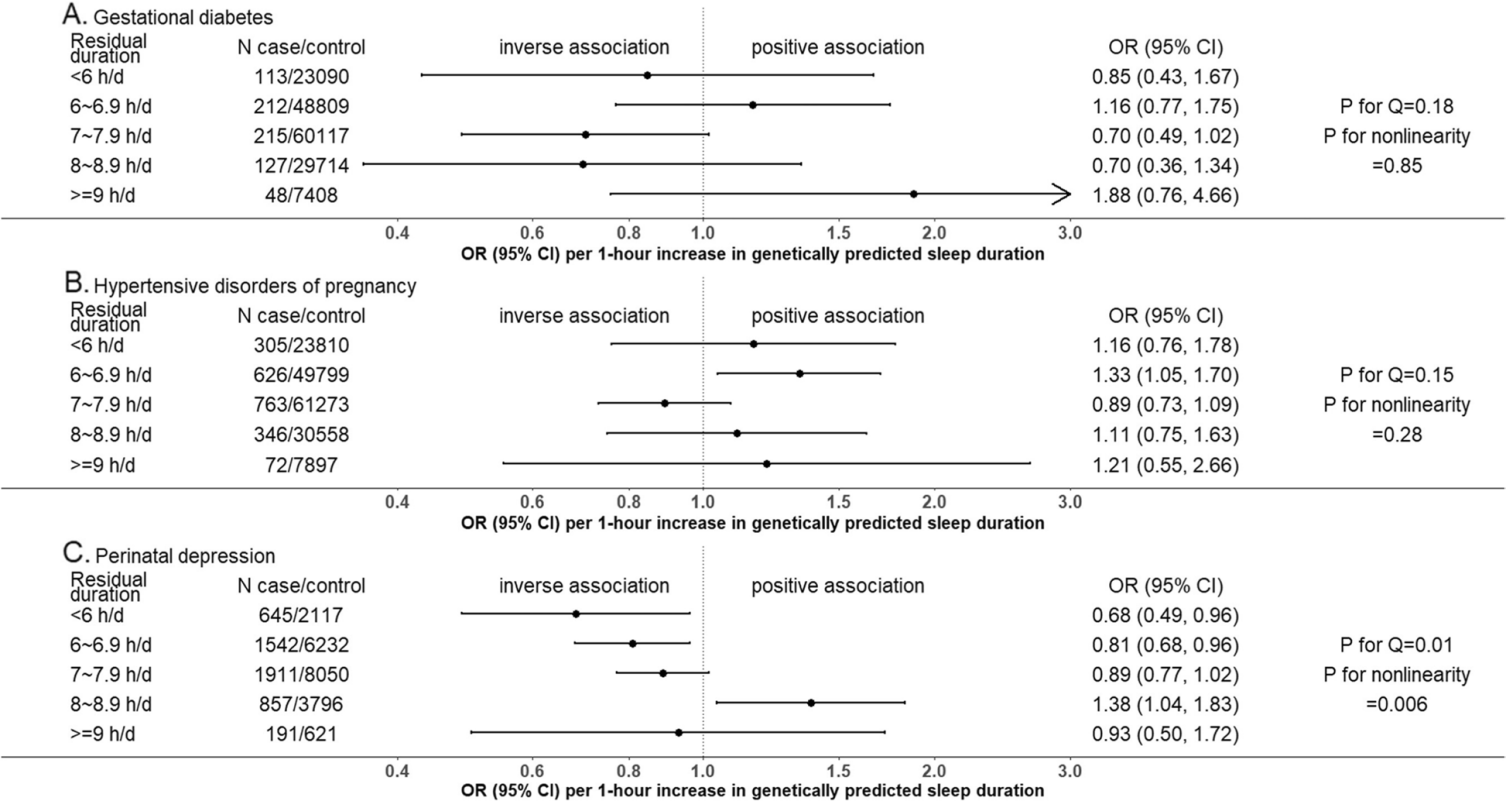
One-sample MR estimates of effects of sleep duration on pregnancy-related outcomes in five groups in UK Biobank women. We present MR estimates of a linear effect of increasing duration on the outcome across the length of residual duration (h/day) covered in each group. Further details about identifying the pattern of nonlinear effects are illustrated in Table 1. *P*-value for Cochran’s Q-statistic testing statistical evidence for between-group heterogeneity. *P*-value for nonlinearity testing statistical evidence whether the MR estimates are changed as the self-reported sleep duration mean increases. Abbreviation: CI, confidence interval; HDP, hypertensive disorders of pregnancy; MR, Mendelian randomization; OR, odds ratio

**Fig. 4:**
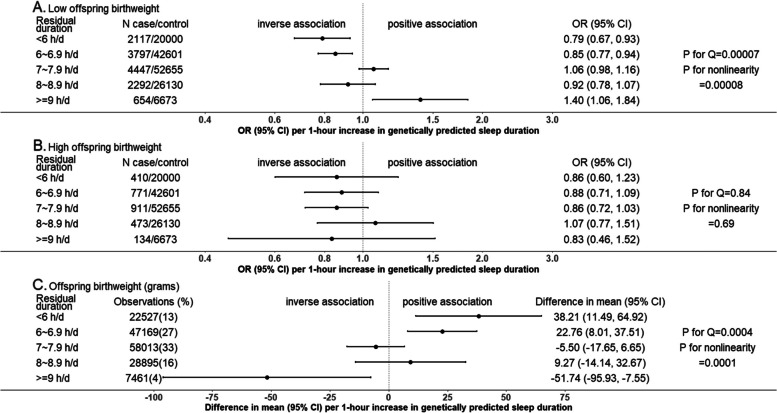
One-sample MR estimates of effects of sleep duration on offspring-related outcomes in five groups in UK Biobank women. We present MR estimates of a linear effect of increasing duration on the outcome across the length of residual duration (h/day) covered in each group. Further details about identifying the pattern of nonlinear effects are illustrated in Table 1. *P*-value for Cochran’s *Q*-statistic testing statistical evidence for between-group heterogeneity. *P*-value for nonlinearity testing statistical evidence whether the MR estimates are changed as the self-reported sleep duration mean increases. Abbreviation: CI, confidence interval; MR, Mendelian randomization; OR, odds ratio

Figures 2, 3 and 4 show the MR linear effects within five sleep duration groups; the differences in directions and magnitudes of the results between groups can be used to describe the pattern of nonlinear effects of sleep duration. There was evidence of nonlinear effects of sleep duration on stillbirth, perinatal depression and low birthweight, with a pattern suggesting that both shorter and longer sleep duration increased risks of stillbirth and low birthweight, with shorter sleep duration increasing perinatal depression (Figs. 2, 3 and 4). The nonlinear effect with mean birthweight was broadly consistent with low birthweight as expected (Fig. 4). For other outcomes, there was no strong evidence of nonlinearity, though we acknowledge that several of the within-group results are imprecise with wide, and hence overlapping confidence intervals. Patterns were generally similar for the analyses with three groups (Additional file 2: Fig S3) [1, 8].

### Two-sample MR exploring linear effects

In two-sample MR, the mean *F*-statistic for 78 sleep duration-IVs in 175,449 women was 15. In MoBa, 0.42% variation of sleep duration in pregnancy was explained by its IV, and one effect allele increase in the IV was associated with 0.008 h/d longer sleep duration (95% confidence interval: 0.003, 0.013, *P*-value = 0.0009, *F*-statistic = 31). Additional file 1: Tables S3 & 7 show summary results for SNP-sleep duration and SNP-outcome associations used in two-sample MR. After adjusting for fetal genotype (only possible in the birth cohorts), SNP-outcome associations with stillbirth, hypertensive disorders of pregnancy, perinatal depression, preterm birth and low birthweight were slightly attenuated; the associations with miscarriage, gestational diabetes, high birthweight and birthweight moved slightly away from the null (Additional file 2: Fig S4).

**Fig. 5:**
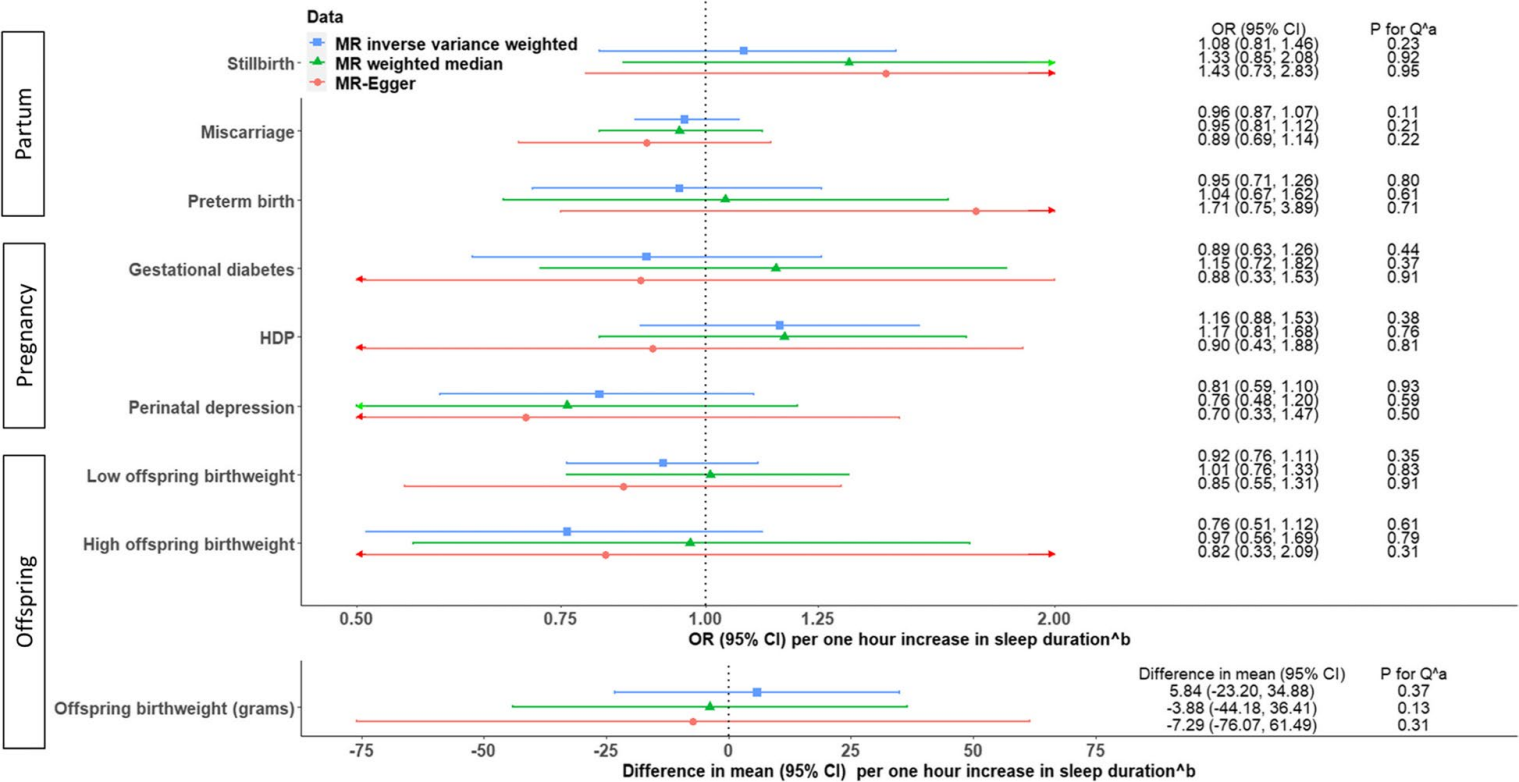
Two-sample MR estimates for linear effects of sleep duration on partum-, pregnancy- and offspring-related outcomes, meta-analysing all cohorts. ^a^*P*-values for Cochran’s Q-statistic testing statistical evidence for between-cohort heterogeneity, in inverse variance weighted, weighted median and MR-Egger, respectively. ^b^Results are ORs for binary outcomes (mean difference in birthweight) per lifetime genetic tendency to 1 h longer during the 24-h duration, with numeric estimates listed in Additional file 1: Table S8. Abbreviations: CI, confidence interval; HDP, hypertensive disorders of pregnancy; MR, Mendelian randomization; OR, odds ratio

The main IVW analyses combining UKB with other cohorts find no strong evidence to support a linear effect across the whole distribution of reported sleep duration of lifetime predisposition to longer average duration on the pregnancy and perinatal outcomes (Fig. 5). However, 95% confidence intervals were wide for most outcomes (Fig. 5). Sensitivity analyses using weighted median and MR-Egger for these outcomes were largely directionally consistent (Fig. 5). Between-SNP heterogeneity for MR analyses was observed for preterm birth, gestational diabetes, hypertensive disorders of pregnancy, perinatal depression, high birthweight and birthweight (Additional file 1: Table S8), but leave-one-out analyses suggested little evidence for a single SNP driving the MR IVW results (Additional file 2: Fig S5-S7). The MR-Egger intercept *P*-values provided little evidence of unbalanced horizontal pleiotropy for any outcome (Additional file 1: Table S8). Across all outcomes, there was little evidence of between-study heterogeneity (all Cochran’s *P*-values >0.1, Fig. 5). IVW analyses using 55 SNPs and 43 SNPs (i.e. those with some evidence of replication) were broadly consistent with those from our main analyses with 78 SNPs (Additional file 2: Fig S8) [2].

### MVreg in MoBa exploring and comparing linear and nonlinear associations

**Fig. 6:**
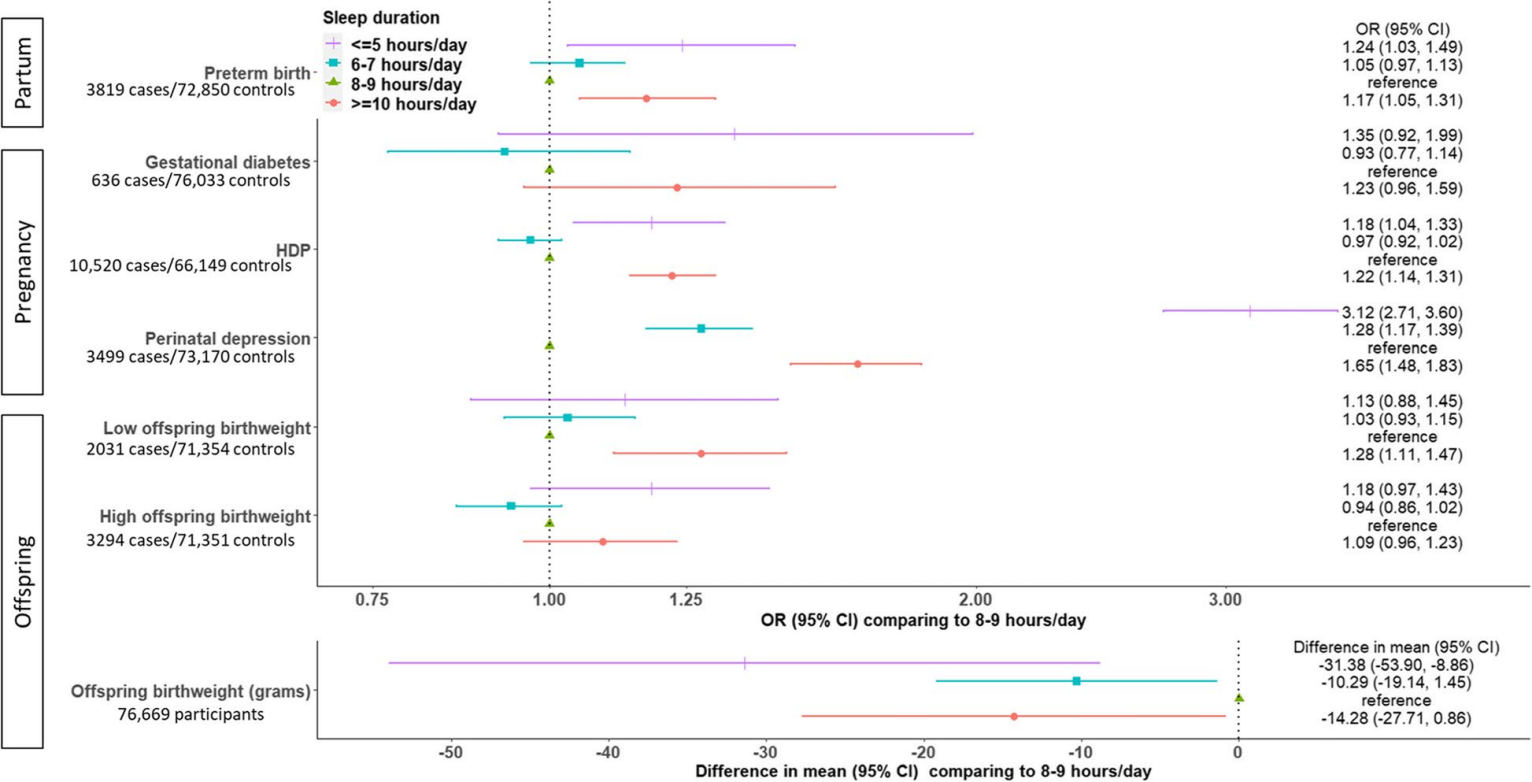
Multivariable regression associations of self-reported sleep duration categories with partum-, pregnancy- and offspring-related outcomes in MoBa. Estimates are from multiple imputation, with numeric results listed in Additional file 1: Table S9. Abbreviations: CI, confidence interval; HDP, hypertensive disorders of pregnancy; MoBa, Norwegian Mother, Father and Child Cohort Study; OR, odds ratio

Nonlinear models fitted data better than linear models across most outcomes (likelihood ratio *P*-values comparing the linear versus nonlinear models = 0.02 to 3.2×10^−52^). The one exception, based on the conventional threshold of <0.05, was gestational diabetes (0.06). Odds of gestational diabetes, hypertensive disorders of pregnancy, perinatal depression, preterm birth, low birthweight and high birthweight were higher in women reporting ≤5 h/d and ≥10 h/d sleep compared with the reference category of 8–9 h, despite some wide confidence intervals including the null (Fig. 6). Amongst them, perinatal depression showed a reverse “J-shaped” nonlinear association with considerably stronger magnitudes of associations than other outcomes (Fig. 6), which is in line with the result in one-sample nonlinear MR in UKB (Fig. 3). Differences in mean offspring birthweight were lower for those reporting ≤5 h/d, 6–7 h/d and ≥10 h/d compared to 8-9 h/d (Fig. 6). Broadly consistent results were observed for low and high birthweight when all eligible participants were included in MVreg (Additional file 2: Fig S9), and for complete records analyses (Additional file 1: Table S9).

## Discussion

In this study, we find MR analyses support nonlinear effects of sleep duration on stillbirth, perinatal depression and low birthweight. With the exception of gestational diabetes, we find statistical evidence for nonlinear associations with all outcomes in MVreg. We have interpreted the MR results as reflecting genetically predicted lifetime sleep duration [18]. We interpreted MVreg results as reflecting associations of sleep duration in pregnancy, though this exposure is likely to correlate strongly with sleep duration before and after pregnancy as demonstrated in a study of 1480 women showing sleep duration 2 years after pregnancy associated with sleep duration at 32 weeks of gestation and 2 months after birth, and with chronic sleep duration [69]. The association between our genetic risk score and sleep duration at 30 weeks of gestation in MoBa indicated that genetic susceptibility to lifetime longer sleep duration is correlated with sleep duration during pregnancy.

Previous observational studies have tended to assume nonlinearity and explored associations of short and long sleep duration with most pregnancy and perinatal outcomes, reporting unfavourable associations of both short and long duration with gestational diabetes, and of short duration with pre-eclampsia and preterm birth [7–10, 12]. Our MVreg analyses in one of the largest studies to date were broadly consistent with these findings. In MVreg, we found evidence of increased risks of perinatal depression with short and long duration, whereas a previous systematic review reported an association with shorter sleep duration only, which is consistent with our one-sample MR analyses [6]. MR analyses also supported increased risks of stillbirth and low birthweight with shorter and longer duration. MR evidence for other outcomes was too imprecise to make reliable conclusions. Furthermore, differences between what have been estimated (linear lifelong effects within groups of residual duration in one-sample MR versus odds of outcomes comparing for self-reported duration categories to a reference category in pregnancy in MVreg) make direct comparisons between our MR results versus our, and other published, MVreg associations difficult. As the sources of bias in two methods (MR and MVreg) differ, where we have consistency between them, this increases our confidence in those consistent results being the causal association [33, 38, 70–72]. The broad consistencies of nonlinear associations of sleep duration with a range of pregnancy and perinatal outcomes between our MVreg and existing literature highlight the need for further MR analyses of potential nonlinear effects in much larger sample sizes with sleep duration measured in pregnancy. The MoBa sample size of >14,000 is too small for nonlinear MR but with further GWAS in that study this might become possible in the future.

The associations of shorter and longer sleep duration with pregnancy and perinatal outcomes may be related to sleep fragmentation, circadian dysrhythmia, insulin resistance and chronic inflammatory [7, 8]. MR analyses support a causal effect of chronic inflammation on depression and anxiety outside of pregnancy [73], and therefore, this is a plausible mechanisms for perinatal depression. Multiple lines of evidence (including MR) demonstrate the positive linear effect of higher maternal glucose on higher birthweight [74, 75]. Therefore, insulin resistance is unlikely to mediate the effect we observe on low birthweight, but it is possible that inflammation has a role here [76].

### Strengths and limitations

The main strengths of our study include that (1) as far as we are aware it is the first time that MR has been used to explore potential linear and nonlinear effects of lifetime sleep duration on pregnancy and perinatal outcomes; (2) we conducted confounder-adjusted MVreg of sleep duration in pregnancy in MoBa, with a larger sample size than most previous studies; and (3) we explored a range of pregnancy and perinatal outcomes.

Our MR analyses may be vulnerable to weak instrument bias, especially within some sleep duration groups in one-sample nonlinear MR which is expected to be biased towards the confounded MVreg result [58]. We used a genetic risk score to minimize the contribution of weak IVs in one-sample MR (with the lowest *F*-statistic=64). Weak instrument bias in our two-sample MR analysis would bias the results towards the null [58]. Our MR analyses may be biased by horizontal pleiotropy, particularly because IVs for sleep duration have also been strongly linked to other sleep traits [2], as well as lifestyle factors such as obesity and alcohol consumption [77]. We explored the potential presence of bias by horizontal pleiotropy with sensitivity analyses for two-sample MR, which are more robust to such bias than IVW [62], but one-sample MR estimates in each stratum could still be prone to horizontal pleiotropy, as methods available for exploring horizontal pleiotropy in one-sample MR would be underpowered in each stratum [63]. We were able to demonstrate that results were not biased by a path via fetal genotype to the outcome, showing similar maternal SNP-outcome associations with and without adjustment for fetal genotype. The MR monotonicity assumption requires that the proposed genetic IVs for longer sleep duration cannot increase sleep duration in some women while decreasing it in others—i.e. women are “compliers” [78]. In nonlinear MR, constant IV-duration effects across all groups is a stronger version of the monotonicity assumption [19]. As we found slightly different genetic risk score-duration associations across groups, our nonlinear MR results should be interpreted cautiously as tests for causal directions rather than as precise estimations of causal effects [78].

Both our MR and MVreg estimates could be vulnerable to selection bias as discussed in detail in other papers [79–81]. UKB participants are better educated and healthier than the general UK adult population [82], and perinatal depression and preterm birth may not be missing at random [28, 83]. Our target population consisted of women who have had at least one pregnancy. In UKB and birth cohorts, we were able to restrict the analyses to ever pregnant women. In FinnGen, the comparison group is all women without the outcome (i.e. including women who have never been pregnant). These are publicly available data that we cannot reanalyse. However, any potential bias resulting from this is unlikely to be substantial given we obtained near-identical results from UKB when we included never pregnant women to those presented here, and results did not substantially differ if we removed FinnGen from our analyses.

Sleep duration was measured via one self-administrated question in UKB and MoBa. Its measurement error in UKB may not bias our MR estimates, as sleep duration is a continuous variable in 1-h units [84]. However, non-differential misclassification of sleep duration categories in MoBa would be expected to bias MVreg towards the null [85]. Further studies using large, actigraphy-based sleep duration data would be required to explore optimal cut-off points for our suggestive nonlinear associations [19]. Moreover, it was ambiguous whether the variable in MoBa represented nocturnal sleep duration or included daytime naps. For our outcomes, there may be misclassification because of the absence of universal testing, assessment via self-report questionnaires, and differences between studies in definitions. Our MVreg analyses for pregnancy outcomes (including gestational diabetes, hypertensive disorders of pregnancy and perinatal depression) in MoBa could be vulnerable to reverse causality, as they were defined based on some information earlier than 30 weeks of the index pregnancy. Furthermore, our MVreg analyses in MoBa could be biased by residual and unmeasured confounding.

## Conclusions

Our study shows that shorter and longer sleep duration increase risks of stillbirth, perinatal depression and low birthweight. Our MVreg analyses support nonlinear over linear effects of sleep duration on all pregnancy and perinatal outcomes, except gestational diabetes. Taken together these findings highlight the need for further MR studies based on larger numbers, particularly of cases for pregnancy and perinatal outcomes. Studies in women from non-White European ethnic background are necessary for understanding the extent to which results generalize to other groups.

## Supplementary Information

**Additional file 1: Table S1**. Results extracted from recent systematic reviews. **Table S2**. Key characteristics of GWAS of self-report sleep duration. **Table S3**. SNP list and female-specific effect estimates of sleep duration SNPs identified in UK Biobank. **Table S4**. Definitions of outcomes. **Table S5**. Details of UKB women by sleep duration groups in one-sample MR. **Table S6**. Characteristics of the women in MoBa by sleep duration categories. **Table S7**. Associations of 78 SNPs with sleep duration and with outcomes in UKB, ALSPAC, BiB, and MoBa. **Table S8**. Two-sample MR estimates for causal effects of sleep duration on the outcomes. **Table S9**. MVreg associations of self-reported sleep duration categories with the outcomes in MoBa.

**Additional file 2: Text S1**. Descriptions of each cohort. **Text S2**. Technical considerations of one-sample MR to explore potential nonlinear effects. **Fig S1**. Flow chart. **Fig S2**. Histogram of residual sleep duration in UKB women. **Fig S3**. One-sample MR estimates of effects of sleep duration on the outcomes in UKB women. **Fig S4**. Associations of 78 SNPs with the outcomes with versus without adjustments of fetal genotypes. **Fig S5**. Leave-one-out analyses in UKB (datasets A on B). **Fig S6**. Leave-one-out analyses in UKB (datasets B on A). **Fig S7**. Leave-one-out analyses in other cohorts. **Fig S8**. Two-sample MR associations of sleep duration with the outcomes using IVW. **Fig S9**. Comparing MVreg associations of sleep duration categories with odds of low/high birthweight using different outcome comparison groups.

## Data Availability

We used both individual participant cohort data and publicly available summary statistics. We present summary statistics that we generated from those individual participant cohort data in Additional file 1: Tables S3 & S7. Full information on how to access UKB data can be found at its website (https://www.ukbiobank.ac.uk/researchers/). All ALSPAC data are available to scientists on request to the ALSPAC Executive via this website (http://www.bristol.ac.uk/alspac/researchers/), which also provides full details and distributions of the ALSPAC study variables. Similarly, data from BiB are available on request to the BiB Executive (https://borninbradford.nhs.uk/research/how-to-access-data/). Data from MoBa are available from the Norwegian Institute of Public Health after application to the MoBa Scientific Management Group (see its website https://www.fhi.no/en/op/data-access-from-health-registries-health-studies-and-biobanks/data-access/applying-for-access-to-data/ for details). Summary statistics from FinnGen are publicly available on its website (https://finngen.gitbook.io/documentation/data-download). All scripts are available on GitHub (https://github.com/MRCIEU/sleep_duration_pregnancy).

## Abbreviations

ALSPAC: Avon Longitudinal Study of Parents and Children
BiB: Born in Bradford
CHARGE: Cohorts for Heart and Aging Research in Genomic Epidemiology
GWAS: Genome-wide association study
h/d: Hours/day
IVs: Instrumental variables
IVW: Inverse variance weighted
MoBa: Norwegian Mother, Father and Child Cohort Study
MR: Mendelian randomization
MVreg: Multivariable regression
SNPs: Single nucleotide polymorphisms
STRATOS: STRengthening Analytical Thinking for Observational Studies
UKB: UK Biobank
